# Exploring the world for longitudinal datasets: data resources for transformative mental health research

**DOI:** 10.1093/ije/dyaf128

**Published:** 2025-07-21

**Authors:** Louise Arseneault, Bridget T Bryan, Thomas Canning, Malaika Okundi, Alice Stephens, Elena Triantafillopoulou, Daniel Yu, Mariana Bolivar, Parisa Mansoori, Lea Milligan, Ed Evans, Grace Gatera

**Affiliations:** Social, Genetic and Developmental Psychiatry Centre, Institute of Psychiatry, Psychology and Neuroscience, King's College London, London, United Kingdom; Social, Genetic and Developmental Psychiatry Centre, Institute of Psychiatry, Psychology and Neuroscience, King's College London, London, United Kingdom; Social, Genetic and Developmental Psychiatry Centre, Institute of Psychiatry, Psychology and Neuroscience, King's College London, London, United Kingdom; Department of Biostatistics and Health Informatics, Institute of Psychiatry, Psychology and Neuroscience, King’s College London, London, United Kingdom; Social, Genetic and Developmental Psychiatry Centre, Institute of Psychiatry, Psychology and Neuroscience, King's College London, London, United Kingdom; Social, Genetic and Developmental Psychiatry Centre, Institute of Psychiatry, Psychology and Neuroscience, King's College London, London, United Kingdom; National Centre for Social Research, London, United Kingdom; Social, Genetic and Developmental Psychiatry Centre, Institute of Psychiatry, Psychology and Neuroscience, King's College London, London, United Kingdom; Social, Genetic and Developmental Psychiatry Centre, Institute of Psychiatry, Psychology and Neuroscience, King's College London, London, United Kingdom; Department of Psychology, University of Bath, Bath, United Kingdom; MQ Mental Health Research, London, United Kingdom; MQ Mental Health Research, London, United Kingdom; MQ Mental Health Research, London, United Kingdom; Open Data Institute, London, United Kingdom; Mental Health Team, Wellcome Trust, London, United Kingdom

## Introduction 

Key to our understanding of mental health conditions are longitudinal data that allow the examination of developmental patterns and factors that precipitate, increase, buffer or parallel mental ill health. A priority is to maximize the use of the data already collected by existing studies, given their research and financial value. A global effort to seek out longitudinal datasets worldwide could serve as a catalyst for boosting the uptake of data and maximizing the value for money of existing data resources.

For more than half of the population worldwide, the onset of mental health conditions typically occurs before the age of 25 [1–3]. Longitudinal studies that start in the early years of life, prior to the typical onset of mental health conditions, allow for a deeper understanding of temporal trends and causal mechanisms that can explain how environmental, biological, and genetic factors influence mental health conditions. In addition to the data they collect, longitudinal studies are instrumental because of the populations they represent. Groups of individuals at increased risk for mental health conditions are especially valuable for developmental research. Sample size is another consideration; larger groups of individuals enable the exploration of important factors with smaller effect sizes and mitigate the impact of participant attrition across time. Additionally, the granularity of assessment enhances the worth of longitudinal studies by capturing rapid changes during pivotal life stages and transitions. This is especially pertinent in childhood, a foundational period for learning and experimentation, and during adolescence, characterized by sudden physical changes and expanding social networks.

We undertook a global search for existing longitudinal datasets to provide a springboard for innovative mental health research by leveraging the current landscape of longitudinal data resources. We report here an overview of the datasets we identified, their overall richness and areas for enrichment, and observations to consider as we expand longitudinal data resources.

## Mapping longitudinal datasets worldwide

**Figure 1.**
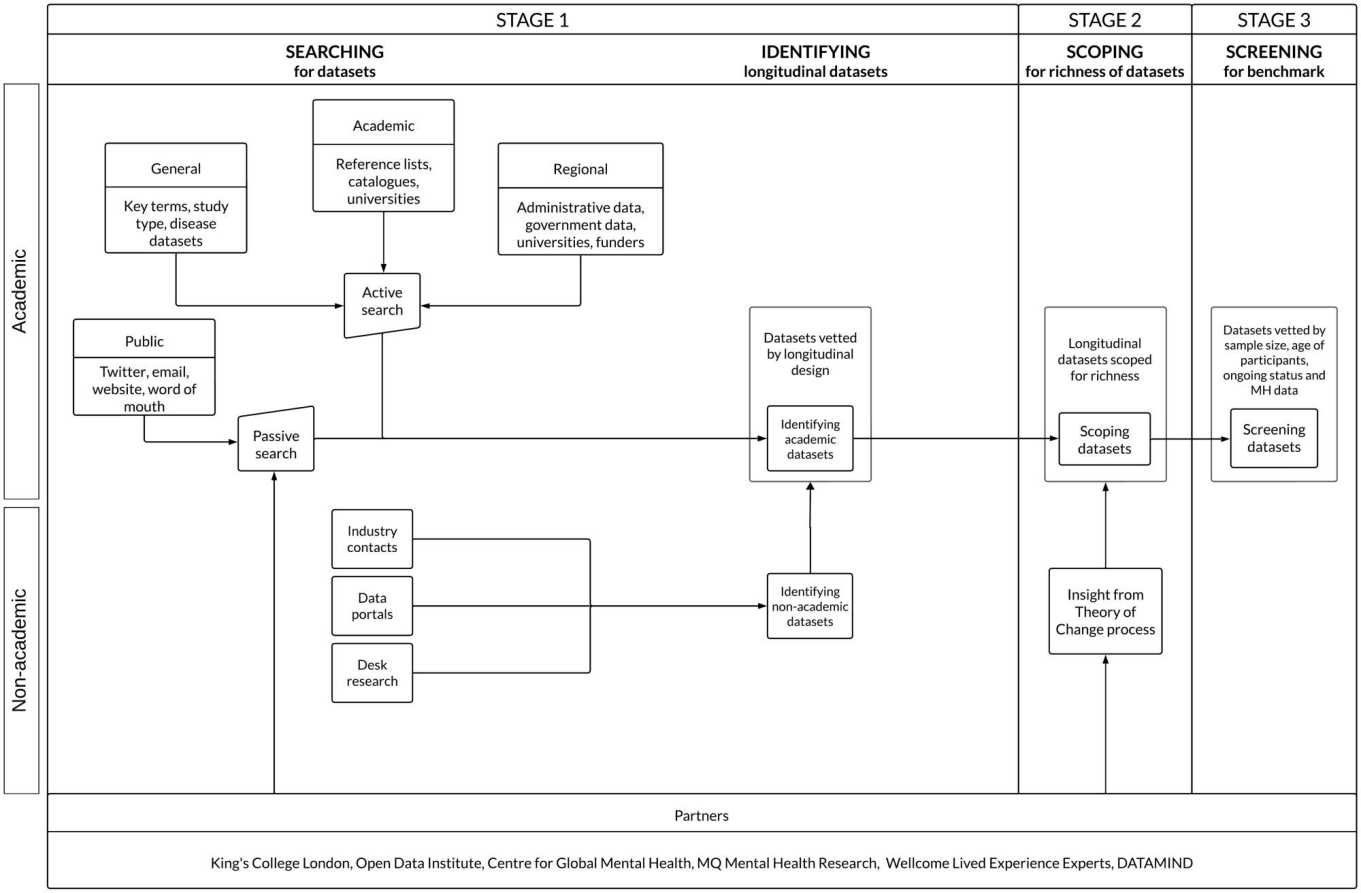
Flowchart presenting the key partners and the stages for landscaping longitudinal datasets worldwide. For datasets in academia, we adopted an active search approach for identifying longitudinal datasets which involved looking through repositories containing information about multiple datasets, such as academic journals and global consortia. Examples of repositories include the Maelstrom Research platform, the International Network for the Demographic Evaluation of Populations and Their Health (INDEPTH) Data Repository, and the Health Data Research Gateway. We screened academic journals such as the International Journal of Epidemiology and Advances in Life Course Research for cohort profile papers. We also pulled datasets from global constoria, such as the International Household Survey Network, the Asia Pacific Cohort Studies Collaboration, and the International Hundred Thousand Cohorts Consortium. We also adopted a passive search approach which involved receiving information from individuals across the world in the mental health and epidemiology communities regarding datasets and repositories. For datasets outside academia, the team engaged in three complementary approaches. First, approaching industry contacts to identify relevant datasets and discuss barriers to sharing non-academic data. Second, searching open data portals using an automated strategy. Third, using desk research to focus on the most promising types of non-academic datasets previously identified.

Figure 1 represents our process for identifying all longitudinal datasets from across the world, including those that delved into mental health and other research topics including physical health or socioeconomic factors. We examined datasets without mental health data to date, as well as those rich in mental health data. We considered datasets to be longitudinal if they spanned a minimum of two waves of data collection. We surveyed cohorts (which follow a group of individuals over time) and household/community panels (which use the household or the community as the unit of analysis over time). We also inspected data collected routinely for non-research purposes (e.g. health registries, surveillance panels) and biobanks (i.e. repositories of human biological samples) with multiple waves of data collection. We included datasets in any language. We excluded cross-sectional studies and randomized controlled trials.

We explored all identified longitudinal datasets to unravel the richness of their data and their populations. This scoping exercise was guided by our experience reviewing thousands of longitudinal datasets and by the input from stakeholders who took part in a Theory of Change (ToC) process. ToC processes are used to define the steps that need to happen for a desired outcome to be achieved [4–6]. For this project, the ToC process informed how longitudinal datasets could be enriched to facilitate transformative mental health research. We adopted a co-production methodology by involving individuals with lived experience (LE) of mental health conditions, as well as researchers and policymakers from various countries and demographic backgrounds. Their input was captured through a pre-workshop package, an online workshop discussion, and a post-workshop meeting. The ToC process occurred alongside the search, allowing the findings and insights from each process to inform the other [7].

We screened longitudinal datasets according to benchmark criteria proposed by the Wellcome Trust. The benchmarks were that a longitudinal dataset:

1. Consists of at least 8000 participants at inception;
2. Has collected, or has the potential to collect, data from participants at some point between the ages of 14 and 30;
3. Has collected data at least every 3 years, or can contact participants to invite them for more frequent data collection;
4. Has collected data in the last 3 years, has plans to collect new data, or is in contact with participants.

## The abundance of longitudinal datasets

**Figure 2.**
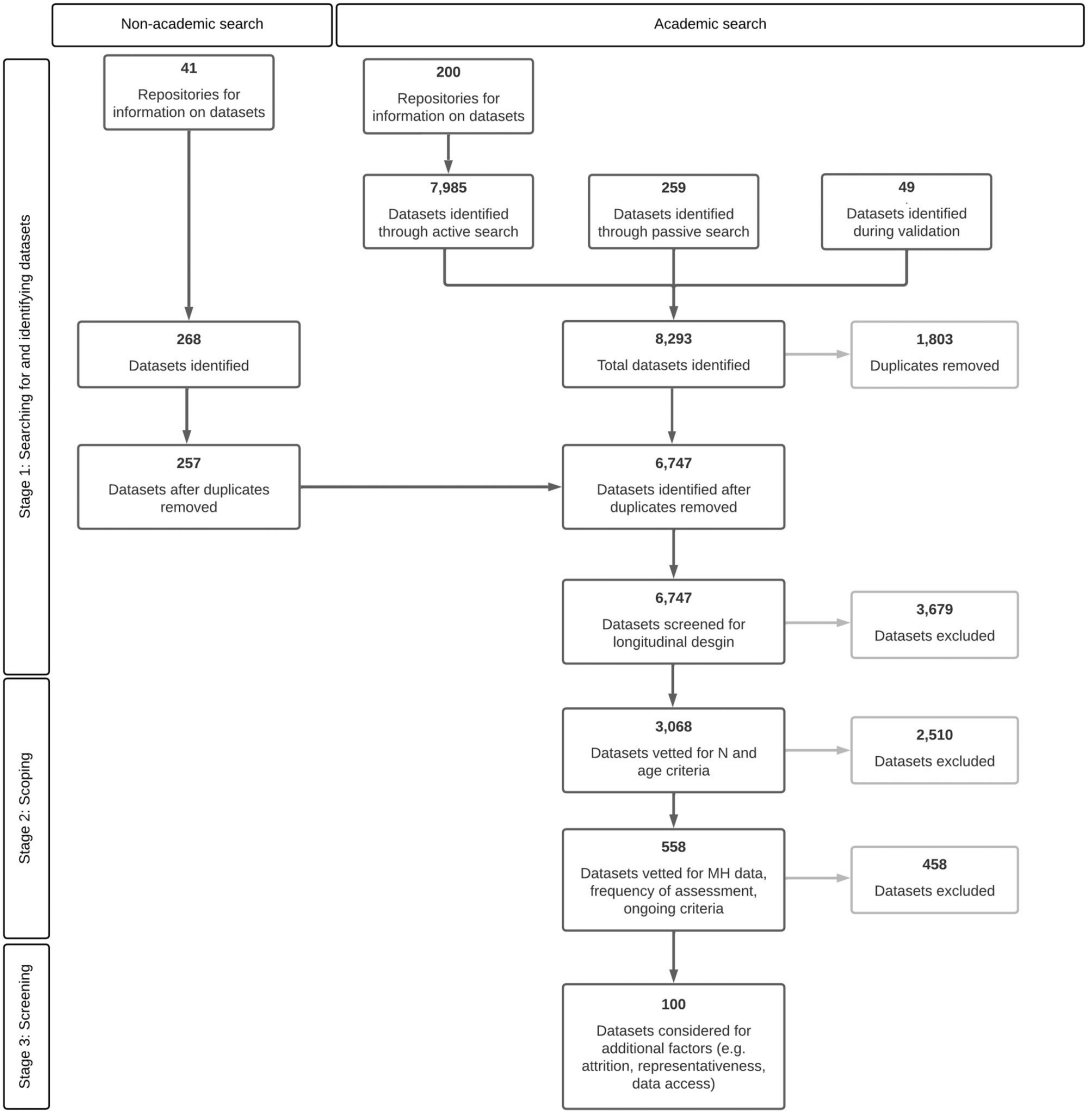
Flowchart reporting the number of datasets processed during the landscaping stages.

**Figure 3.**
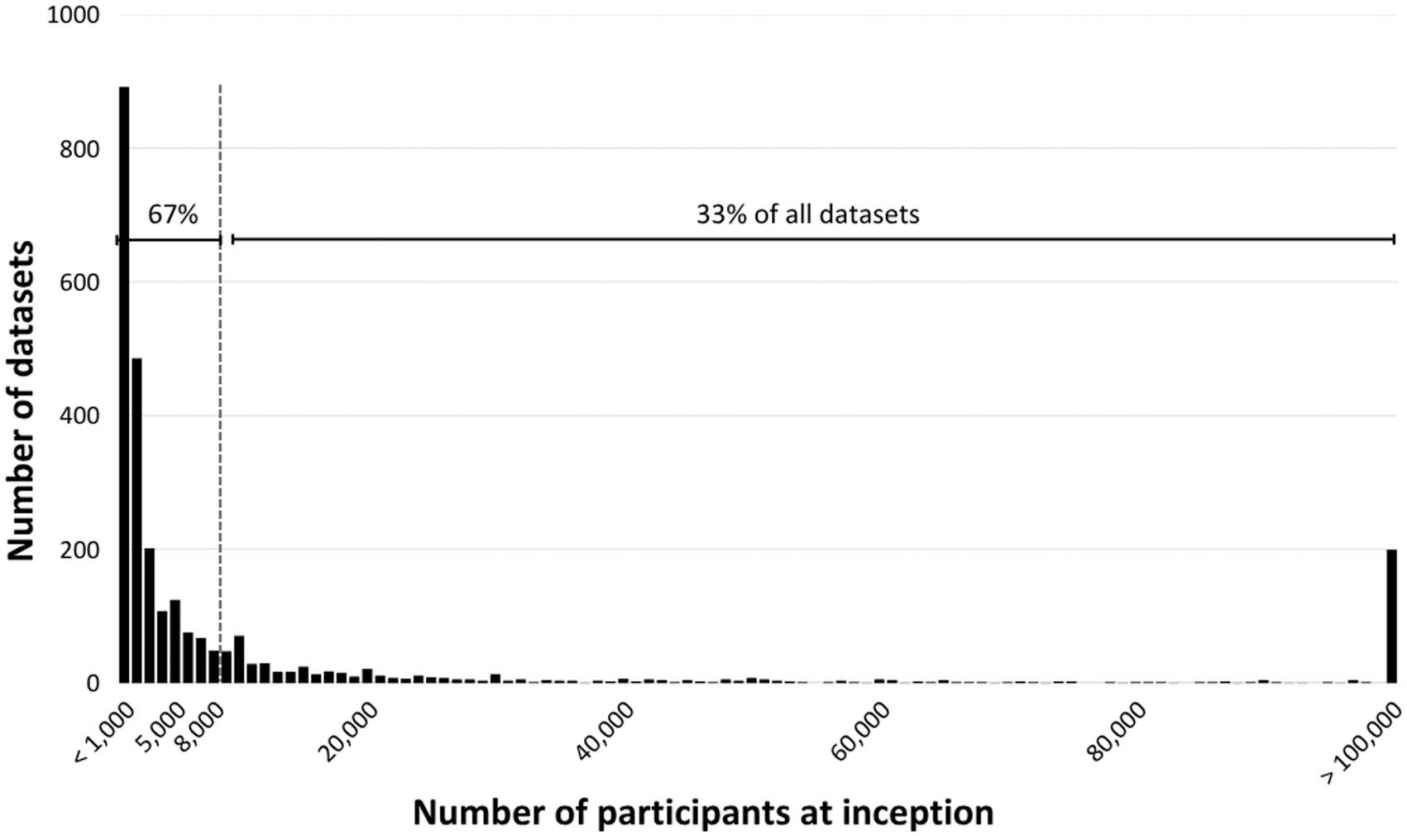
Number of participants at inception for all identified longitudinal datasets.

From the 6747 unique datasets we screened, we identified over 3000 longitudinal datasets from around the world (Fig. 2). The large number of datasets that we identified during our comprehensive investigation include data collected from over 700 million individuals in 146 countries. A total of 100 longitudinal initiatives were ongoing, based on large samples, and had granular assessments of young populations. Most datasets enrolled fewer than 8000 participants at their inception (Fig. 3). However, a subset stands out with exceptionally large sample sizes. These large data sources are often based on routinely collected data from health and government records, or multi-site initiatives. Our scoping exercise underscores the wealth and range of existing longitudinal data for exploring developmental patterns of mental health conditions.

## Longitudinal data resources are not equally distributed across the world

Our investigation revealed an unequal distribution of longitudinal datasets worldwide. A considerable proportion of the identified datasets originated from Europe (40%). This region encompasses 44 primarily affluent countries, distinguishing it from other regions with a greater number of low- and middle-income countries (LMICs). For instance, Africa, the Middle East, and Asia include 104 countries that are primarily LMICs and generated 25% of the overall identified longitudinal resources. In contrast, the Americas, which encompass 36 countries and represent some LMICs, contributed an equivalent of 25% of the total identified longitudinal datasets. We noted a few longitudinal resources in LMICs, which disproportionately face economic and geopolitical challenges. The concentration of study populations and research infrastructure in wealthy Western countries limits the generalizability of mental health research findings [8, 9].

## Mental health has rarely been a priority for large-scale longitudinal research

**Figure 4.**
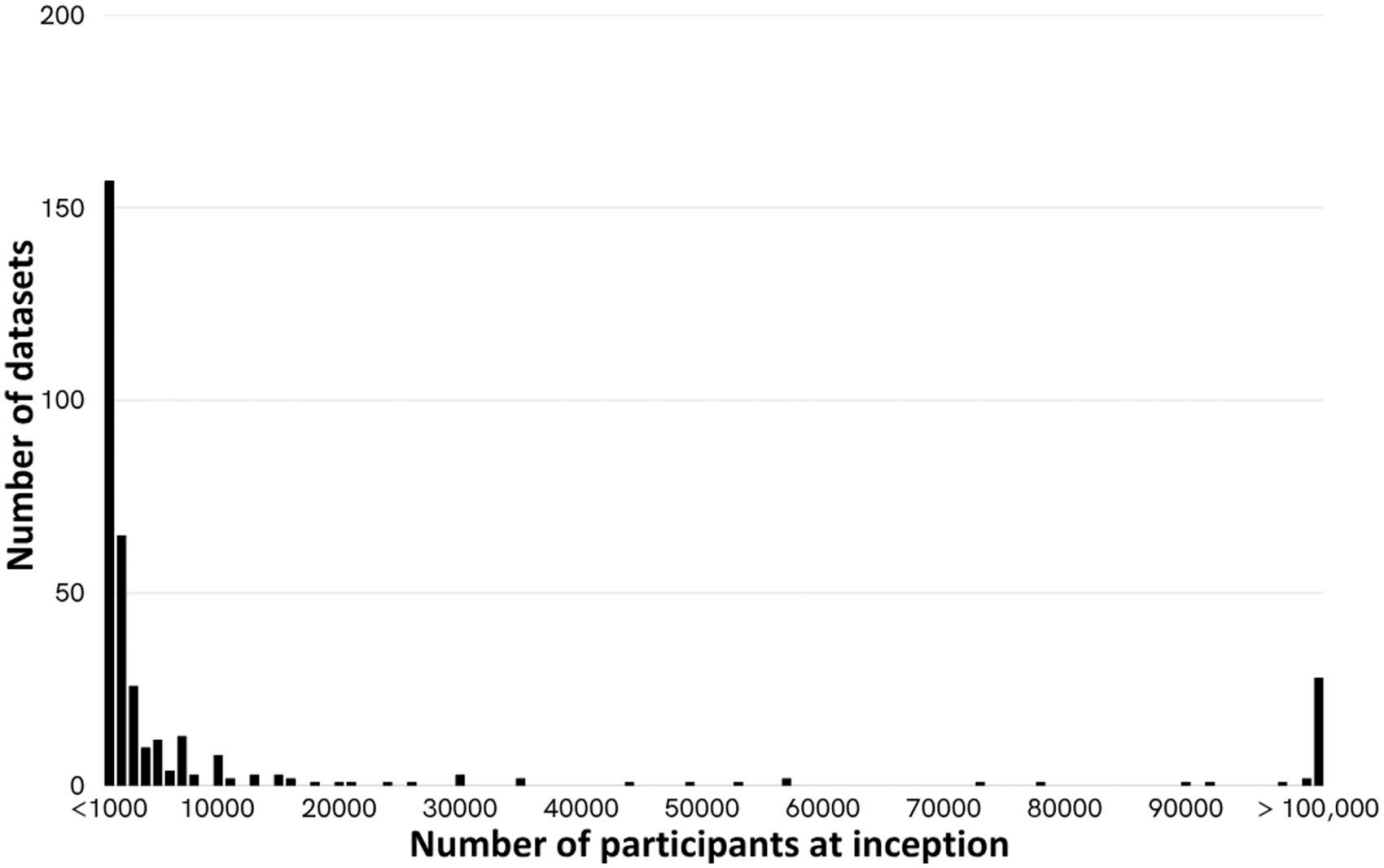
Number of participants at inception for longitudinal datasets focused on mental health (*N* = 358).

Mental health has rarely taken precedence for large longitudinal initiatives, and those with a specific focus on mental health tend to be smaller in scale (Fig. 4). More than 75% of the datasets that focus on mental health have enrolled fewer than 5000 participants. Several factors may contribute to this pattern. Firstly, mental health may not have been a priority for policy and funding when the studies were established [10, 11], leading to a scarcity of resources dedicated to expansive mental health-focused longitudinal studies. Secondly, the limited evidence-based avenues for transformative mental health interventions result in pathways for action that are less clear than for physical health conditions. This may hinder the motivation for large-scale mental health research. Thirdly, the challenges associated with the assessment of mental health conditions could contribute to the preference for smaller size and more targeted studies. Collectively, these considerations underscore the need for increased focus, resources, and innovative approaches to enhance the integration of mental health assessments within larger-scale longitudinal datasets.

## Richness in longitudinal data worldwide

There are abundant opportunities for mental health research in existing longitudinal datasets. We clustered them into four categories representing domains within the broader landscape of mental health research. This categorization offers a structured framework for researchers and funders to navigate and explore the specific areas where these datasets can contribute.

1. *Richness in mental health measures*: strong measurement of depression and anxiety, valuable resources for psychosis research, consideration of other mental health conditions, and mental health across the life course.
2. *Value in targeted populations*: high-risk groups, under-represented groups, populations from under-represented locations, ageing populations, and value in the long run.
3. *Diversity of data*: factors contributing to mental health conditions, neuroimaging data, wearables and phone apps, biological and genetic data, and routinely collected data.
4. *Mental health embedded in wider context*: mental health within social context, the impact of the coronavirus disease 2019 (COVID-19) pandemic, connectivity between datasets, natural disasters and geopolitical factors, and interventions embedded in longitudinal datasets.

The global distribution of longitudinal datasets across various categories of richness showcases contributions from all geographical regions: Africa, the Middle East, and Asia (7% of total longitudinal datasets from this region), the Americas (15%), Europe and the Pacific (12%), and cross-regions (8%).

## Recruitment of targeted populations is the cornerstone of longitudinal research

The representation of minoritized and marginalized groups was insufficient or absent in most longitudinal datasets. Individuals, such as those from ethnically or racially minoritized groups [12], Lesbian, Gay, Bisexual, and Transgender (LGBT) communities [13], neurodivergent individuals [14, 15], and people experiencing homelessness [16] are at a elevated risk of mental ill health and more likely to be missed during recruitment or lost to follow-up in longitudinal research. As a result, these groups are often under-represented in longitudinal research unless explicitly targeted. Recruitment in longitudinal research ought to be set out with a commitment to maintaining diverse and representative samples, recognizing that inclusive datasets are fundamental for generating findings that resonate with the broader population. This approach not only safeguards against biases for interpreting mental health trends but also fosters an equitable understanding of the factors influencing mental health outcomes across diverse groups.

## Lack of gold standards for mental health measurement

Our review highlighted variation in methods and instruments employed to measure mental health conditions, including standardized and tailor-made measures. There are many ways to assess mental health conditions as they are contingent upon various considerations such as the unique characteristics of the population when selecting instruments. It is important that researchers opt for measures that are most appropriate for participants’ developmental stage and are cognizant of contextual nuances including cultural features. Copyright and language translation requirements can also prevent the use of shared measures across borders and different languages. There is no one-size-fits-all approach to assessing mental health conditions and variation in measurement offers the flexibility required to appropriately assess mental health across diverse contexts and populations.

However, this variation in measurement approaches has also, in part, arisen due to mental health often being treated as an afterthought. Most datasets identified in our search did not focus on mental health, such that the lack of a unified and standardized approach to measuring mental health may have led to difficulties in selecting high-quality, widely used measures of mental health. The diversity in measures used in existing datasets poses challenges in comparing and synthesizing findings across studies and limits the generalizability and robustness of mental health research outcomes.

## Engagement with stakeholders outside academia is scarce

Our review indicates a lack of longitudinal datasets that have incorporated involvement from LE experts (LEEs), communities, and healthcare service users when developing their program of research. The active involvement of the community and collaboration with LEEs contribute significantly to ensuring that longitudinal research is relevant to those who experience mental health conditions [17, 18]. This engagement enhances the applicability of research findings and supports the development of clinical practices that are sensitive to the real-world needs and experiences of individuals dealing with mental health challenges. While there is an increasing recognition of the importance of such involvement and engagement, their absence remains common in longitudinal research [19–21]. Expanding the involvement of LEEs, communities, and service users to longitudinal frameworks can contribute to more holistic, patient-centred, and culturally sensitive approaches to mental health research and intervention strategies [22].

Some recommendations emerged from our search, and they might provide useful insight as the field moves forward.

## Decentralizing longitudinal resources

A prudent consideration as we reflect on the richness of longitudinal datasets is the importance of studies that avoid covering every aspect of human development to optimize the value of a single dataset. The benefits of collecting a wealth of data from one sample should be weighed against significant drawbacks. Overburdening participants by collecting excessive data at frequent intervals may negatively affect participation rates and data quality over time. Instead of pursuing an exhaustive approach with individual studies, researchers and funders might explore the benefits of optimizing the value of groups of particularly promising studies. This can be achieved through concerted efforts in, for example, maximizing sample size, sharing measurement tools, or expanding the geographical location of participants. The collaborative development of consortia for longitudinal initiatives offers important advantages. These initiatives can work together, pooling expertise and data resources when synergies arise and proving especially beneficial for mental health research.

Furthermore, recognizing the potential benefits of longitudinal research in diverse settings and cultures, it becomes imperative to foster cross-national initiatives that support the development and maintenance of longitudinal resources in LMICs. These initiatives can provide vital assistance to researchers in these nations navigating the complexities of collecting and sustaining data. By fostering collaboration and sharing expertise, these cross-national efforts contribute to a more inclusive and comprehensive understanding of temporal trends and patterns across diverse global contexts. This collaborative approach acknowledges the potential richness of data that may emerge from regions facing unique challenges and underscores the importance of global cooperation in advancing longitudinal research. Of utmost importance is ensuring that international collaboration is encouraged by funding schemes while leadership is shared equitably with the teams on site.

## Enriching existing data resources

There are clear benefits to capitalizing on the richness of existing datasets rather than investing in the creation of new longitudinal initiatives. Encouraging researchers to build upon the strengths of established datasets is a strategic approach that can foster collaboration across datasets to maximize the potential for mental health research. We did not find one single ideal source of data for comprehensive mental health research. By enriching groups of existing datasets that complement each other, researchers and funders can increase the value of data already collected by joining forces. This collaborative approach optimizes the efficiency of data collection and utilization, and fosters a more comprehensive understanding of mental health across diverse contexts and using different methods.

## Prioritizing mental health and measurement

There is a compelling need for the mental health research community to prioritize the consolidation and refinement of measurement tools to allow international comparisons across samples and cultures. This involves not only considering the diverse needs of different populations and contexts, but also establishing common standards and frameworks to enhance the consistency and comparability of mental health assessments. Decisions about how to assess mental health conditions should be rooted in careful methodological work that rigorously tests the adequacy of instruments. The complexity of existing measures poses challenges in reconciling findings, harmonizing data, and establishing international collaborations. Furthermore, investing in methodological work is imperative to verify whether measures collected during a different era are suitably comparable. This commitment to methodological rigour will enhance the quality and reliability of mental health research and contribute to the development of more standardized and comparable measurement tools for the future.

The scarcity of emphasis on mental health within large-scale longitudinal initiatives may reflect a notable gap in research priorities and highlight the need for more concerted efforts to incorporate mental health considerations into the fabric of extensive and comprehensive longitudinal studies. The limited representation of mental health-focused datasets emphasizes the urgency of considering mental health within the broader context of longitudinal research given its profound contribution to individual and public health outcomes. Our search indicates an increasing emphasis on mental health, which we hope will continue.

## Making longitudinal data accessible

By carefully considering and optimizing data management and access, researchers and funders can enhance the uptake and overall impact and innovation of longitudinal data. A strategic approach would ensure that data are not only made available to a wide pool of researchers but are also easily accessible and effectively utilized for advancing transformative research in the field of mental health. Data can be made more accessible, more reusable, and more valuable by following guidance on responsible data stewardship [23], increasing trust in data [24], and creating sustainable data ecosystems [25].

Efforts to enhance the discoverability of longitudinal datasets, especially those from diverse cultural and linguistic backgrounds, could also lead to notable advancements in our global understanding of mental health conditions. Developing unified platforms to find information about datasets will also improve data discoverability and facilitate transformative mental health research. This is especially relevant for datasets based in LMICs where collaborations and interactions are more challenging, and countries where language can be a barrier for accessing information and data [26]. There could be major gains in making longitudinal datasets in LMICs and non-English speaking countries more discoverable for a more global appreciation of mental health conditions embedded in social and cultural contexts.

## Limitations of our search strategy

Despite our robust strategy for scoping longitudinal datasets, we must acknowledge its limitations. We cannot claim certainty in having found every possible longitudinal dataset around the world. Our confidence in having identified the most pertinent datasets for mental health research stems from the thoroughness of our search, feedback from extensive networks within the field, and active engagement with researchers and data custodians on social media platforms. The substantial number of datasets identified during the project is an indicator of the strategy's effectiveness in uncovering longitudinal datasets globally. We find reassurance in the diversity of datasets we identified, which encompass the full range of LMICs, various geographical regions, different foci, and affiliations with diverse institutions. Furthermore, mapping the globe for longitudinal datasets and assessing their value is an inherently imprecise endeavour. The estimates presented in this article are not absolute but are aimed at fostering an understanding of the global resources available for mental health research.

Our findings are a snapshot of a point in time. Although longitudinal research is not a fast-moving field and is known for its prolonged development, new initiatives continuously emerge and close down. The decision to openly share the list of identified longitudinal datasets was motivated by the desire to enhance data discoverability and to encourage ongoing collaboration with the research community for accuracy and updates. While our conclusions are likely to remain meaningful and relevant for some time, the evolving landscape of longitudinal data resources will necessitate regular amendments to stay current.

## Conclusion

Our comprehensive global search for longitudinal datasets and thorough analysis of their potential for mental health research unveiled important opportunities for researchers and funders to enhance our understanding of mental health conditions. Adopting a coordinated approach to funding longitudinal studies and a steadfast commitment to enhancing the discoverability and accessibility of existing datasets will optimize the investment of funds, time, and resources made thus far.

## Data Availability

Data from this project is available on https://atlaslongitudinaldatasets.ac.uk/. For the purpose of Open Access, the authors have applied a CC BY public copyright license to any Author Accepted Manuscript version arising from this submission. The views expressed are those of the authors and not necessarily those of the Wellcome Trust or King’s College London.
